# Construction of a mitochondrial dysfunction related signature of diagnosed model to obstructive sleep apnea

**DOI:** 10.3389/fgene.2022.1056691

**Published:** 2022-11-21

**Authors:** Qian Liu, Tao Hao, Lei Li, Daqi Huang, Ze Lin, Yipeng Fang, Dong Wang, Xin Zhang

**Affiliations:** ^1^ Shantou University Medical College, Shantou, China; ^2^ Department of Cardiology, The Affiliated Hospital of Binzhou Medical University, Binzhou, Shandong Province, China; ^3^ Department of General Surgery, The First Affiliated Hospital of Jinan University, Guangzhou, China; ^4^ Laboratory of Molecular Cardiology, The First Affiliated Hospital of Shantou University Medical College, Shantou, China; ^5^ Laboratory of Medical Molecular Imaging, Th e First Affiliated Hospital of Shantou University Medical College, Shantou, China

**Keywords:** mitochondrial dysfunction, obstructive sleep apnea, immunocyte infiltration, bioinformatic analysis, gene signature

## Abstract

**Background:** The molecular mechanisms underlying obstructive sleep apnea (OSA) and its comorbidities may involve mitochondrial dysfunction. However, very little is known about the relationships between mitochondrial dysfunction-related genes and OSA.

**Methods:** Mitochondrial dysfunction-related differentially expressed genes (DEGs) between OSA and control adipose tissue samples were identified using data from the Gene Expression Omnibus database and information on mitochondrial dysfunction-related genes from the GeneCards database. A mitochondrial dysfunction-related signature of diagnostic model was established using least absolute shrinkage and selection operator Cox regression and then verified. Additionally, consensus clustering algorithms were used to conduct an unsupervised cluster analysis. A protein–protein interaction network of the DEGs between the mitochondrial dysfunction-related clusters was constructed using STRING database and the hub genes were identified. Functional analyses, including Gene Ontology (GO) analysis, Kyoto Encyclopedia of Genes and Genomes (KEGG) analysis, gene set enrichment analysis (GSEA), and gene set variation analysis (GSVA), were conducted to explore the mechanisms involved in mitochondrial dysfunction in OSA. Immune cell infiltration analyses were conducted using CIBERSORT and single-sample GSEA (ssGSEA).

**Results:** we established mitochondrial dysfunction related four-gene signature of diagnostic model consisted of *NPR3, PDIA3, SLPI, ERAP2*, and which could easily distinguish between OSA patients and controls. In addition, based on mitochondrial dysfunction-related gene expression, we identified two clusters among all the samples and three clusters among the OSA samples. A total of 10 hub genes were selected from the PPI network of DEGs between the two mitochondrial dysfunction-related clusters. There were correlations between the 10 hub genes and the 4 diagnostic genes. Enrichment analyses suggested that autophagy, inflammation pathways, and immune pathways are crucial in mitochondrial dysfunction in OSA. Plasma cells and M0 and M1 macrophages were significantly different between the OSA and control samples, while several immune cell types, especially T cells (γ/δ T cells, natural killer T cells, regulatory T cells, and type 17 T helper cells), were significantly different among mitochondrial dysfunction-related clusters of OSA samples.

**Conclusion:** A novel mitochondrial dysfunction-related four-gen signature of diagnostic model was built. The genes are potential biomarkers for OSA and may play important roles in the development of OSA complications.

## 1 Introduction

Obstructive sleep apnea syndrome (OSA), a growing health concern that affects nearly one billion people worldwide, is an independent risk factor for cardiovascular and metabolic diseases, but is highly underdiagnosed (Arnaud et al., 2020). Continuous positive airway pressure is highly effective at improving symptoms but cannot reduce the occurrence of comorbidities. The use of biomarkers has been strongly recommended, as the condition often goes undiagnosed because patients remain oblivious to the severity of OSA and its complications (Wang et al., 2022). Therefore, it is an urgent task to find indicators for early diagnosis of OSA and decipher the molecular pathways involved in OSA and its complications in order to ensure earlier treatment and prevent complications.

The physiologic changes in OSA are vast and involve complex mechanisms which play a role in the pathogenesis of cardiovascular and metabolic disorders. Chronic intermittent hypoxia (CIH) is the most deleterious feature of OSA, as it can lead to oxidative damage in every organ (Shan et al., 2007). CIH can suppress mitochondrial function and lead to the generation of reactive oxygen species (ROS) (Wang et al., 2010; Huang et al., 2014; Zhao et al., 2019; Lin et al., 2021; Song et al., 2022). As mitochondrial status is important for the metabolic function of all organs, mitochondrial dysfunction at the cellular level that can affect systemic metabolic balance can significantly contribute to many diseases and have been defined as classical a hallmark of many diseases (Srinivasan et al., 2017; Chapman et al., 2019). Mitochondrial dysfunction is a basic mechanism in inflammation-related non-communicable diseases (Hernandez-Aguilera et al., 2013). The wide acceptance of mitochondrial dysfunction as a correlated factor of Parkinson’s disease (Rocha et al., 2018), cardiovascular diseases (Vásquez-Trincado et al., 2016), diabetic kidney disease (Wei and Szeto 2019) and other numerous diseases (Kasapoğlu and Seli 2020; Yapa et al., 2021) has led to the presupposition that mitochondrial dysfunction markers are associated with OSA. Although various microRNAs and proteins (and their genes) have been reported to be involved in OSA (Li et al., 2017; Cao et al., 2021; Shi et al., 2021; Tang et al., 2021), the effects of OSA on genes and pathways remain largely unknown, especially regarding mitochondrial dysfunction.

Previous studies have suggested that mitochondrial dysfunction represents the molecular mechanism underlying OSA and its comorbidities. First, sleep disorders are prevalent in individuals with mitochondrial disorders; the clinical features of the mitochondrial dysfunction affect the type of sleep disturbance (Brunetti et al., 2021). Second, mitochondrial DNA (mtDNA) copy number is significantly reduced in patients with OSA, and it is a reliable biomarker for predicting cardiovascular risk in patients with OSA (Kim et al., 2014). Third, Banxia-Houpu decoction reduced CIH-induced heart damage by regulating mitochondrial function (Song et al., 2022). Fourth, attenuating mitochondria-dependent apoptosis has been suggested as a novel adjunct strategy for ameliorating OSA-induced neurocognitive impairment (Xu et al., 2021). Fifth, mitochondrial dysfunction and the oxidative stress were found to be involved in genioglossus muscle injuries in OSA with obesity, which may provide therapeutic targets for use in OSA with obesity (Chen et al., 2021). Lastly, research has identified certain proteins associated with CIH, and some may serve as novel biomarkers for OSA and related disorders, such as acute coronary syndrome (Shi et al., 2021) and Alzheimer’s disease (Wu et al., 2021). In conclusion, patients with OSA exhibit several mitochondrial gene mutations, deletions, and some mitochondrial dysfunction indexes. Unfortunately, little has been reported whether mitochondrial dysfunction related genes and pathways could be used as clinical biomarkers of OSA susceptibility and severity so far.

In the present study, a four-gene (*NPR3, PDIA3, SLPI,* and *ERAP*2) diagnostic model was built to diagnose OSA based on mitochondrial dysfunction-related gene expression. The genes are potential biomarkers and therapeutic targets for use in OSA. Consensus clustering of all the samples (OSA and control) was used to identify two mitochondrial dysfunction-related clusters (A and B). Furthermore, to investigate the underlying biological functions of the clusters, we identified 106 differentially expressed genes (DEGs) between clusters A and B and conducted functional enrichment analyses of these DEGs. A protein–protein interaction (PPI) network of the DEGs was constructed using the STRING database. Thereafter, immune cell infiltration was evaluated using both CIBERSORT and single-sample gene set enrichment analysis (ssGSEA). In addition, the correlations between the four diagnostic genes and immune cell infiltration were calculated. To our knowledge, this is the first study to integrate bioinformatics analyses in order to identify the key mitochondrial dysfunction-related genes and pathways, and the degree of immune cell infiltration, in OSA. These genes and pathways may facilitate our understanding of the molecular mechanism of OSA and further provide evidence for early diagnosis, prevention, and treatment of this disease.

## 2 Methods

### 2.1 Data sources and processing

Two microarray datasets [GSE135917 (Gharib et al., 2020) and GSE38792 (Gharib et al., 2013)] on adipose tissue samples from OSA patients and controls were downloaded from the Gene Expression Omnibus (GEO) database. The sequencing platform was GPL96 (HG-U133A)] Affymetrix (Supplementary Table S1). Data of 58 OSA patients and 8 controls from GSE135917, and 10 OSA patients and 8 controls from GSE38792, were analyzed in our study. The datasets were log2 transformed and normalized using the SVA R package. The expression distribution before and after normalization was visualized using boxplots (Supplementary Figure S1).

### 2.2 Analysis of differentially expressed genes and mitochondrial dysfunction-related genes

The limma R package (Ritchie et al., 2015) was used to conduct a DEG analysis comparing the OSA and control samples. The genes with |log(fold change [FC])|>1 and *p* < 0.05 were identified as DEGs. The RCircos R package (Zhang et al., 2013) was used to map the chromosomal location of the genes.

A total of 8334 mitochondrial dysfunction-related genes were then downloaded from the GeneCards database (https://www.genecards.org/) (Safran et al., 2010) using the key words *“mitochondrial dysfunction”*. The mitochondrial dysfunction-related DEGs were then identified.

### 2.3 Correlation analysis among genes

Pearson correlations between pairs of genes were calculated. The GGplot2 R package was used to construct scatter plots of the expression correlations between pairs of genes that met the criteria and to fit correlation curves. The criteria for significant correlation comprised absolute correlation coefficient value >0.5 and *p* < 0.05.

### 2.4 Establishment of diagnostic model

Least absolute shrinkage and selection operator (LASSO) Cox regression was used for feature selection and dimensionality reduction in order to generate a gene-based classifier [9]. To verify the diagnostic value of the model, ROC curves of the single genes and the four-gene model were plotted using R package pROC (Robin et al., 2011). A nomogram and decision curve analysis (DCA) curves were used for validation.

### 2.5 Consensus clustering

Using all the OSA and control samples, a consensus clustering analysis of mitochondrial dysfunction-related genes was used to identify distinct mitochondrial dysfunction-related clusters using the k-means clustering algorithm (Sabah et al., 2021). The optimum number of clusters, along with the consistency of clusters, was determined by the consensus clustering algorithm in the ConsensusClusterPlus package (Seiler et al., 2010). A total of 1000 iterations were performed to ensure the stability of the categories. Additionally, using only the OSA samples, consensus clustering was again used to identify distinct mitochondrial dysfunction-related clusters.

### 2.6 Protein–protein interaction network construction

After determining the DEGs between the mitochondrial dysfunction-related clusters (based on all samples), a PPI network of the DEGs was constructed using STRING network version 11.0 and the default confidence threshold of 0.4. The PPI network was exported and then Cytoscape version 3.8.0 was used to calculate the network attributes of each node. Next, cytoHubba version 1.6 was used to identify hub nodes based on the degree of the nodes.

We predicted the miRNAs and transcription factors related to the hub genes using TarBase (Wang et al., 2013) and miRecords (Fornes et al., 2020). Protein-chemical interactions were obtained from the Comparative Toxicogenomics Database (http://ctdbase.org/) (Davis et al., 2021).

### 2.7 Functional enrichment analyses of differentially expressed genes

Using the DEGs between the mitochondrial dysfunction-related clusters (based on all samples), Gene Ontology (GO) enrichment analysis was employed to study the large-scale functional enrichment of the DEGs at three levels: biological process (BP), molecular function (MF) and cellular component (CC) (Ashburner et al., 2000). Kyoto Encyclopedia of Genes and Genomes (KEGG) enrichment analysis was used to identify the biological pathways related to the DEGs (Kanehisa and Goto 2000). The clusterProfiler R package (Wu et al., 2021) was used to perform GO functional annotation for all significant DEGs to identify significantly enriched GO terms. The enrichment results were visualized using the GOplot R package.

Gene set enrichment analysis (GSEA) using data from MSigDB (Liberzon et al., 2015) was employed to identify the significant differences in biological pathways between the high- and low-expression clusters. The “C2.cp.kegg.v7.4.entrez.gmt” (KEGG pathways) gene set was selected as the reference gene set.

Gene set variation analysis (GSVA) using the “c2.cp.kegg.v7.2.symbols.gmt” (KEGG pathways) and “h.all.v7.2.symbols.gmt” (Hallmark pathways) gene sets (Hanzelmann et al., 2013) was employed to identify the significant differences in biological pathways between the mitochondrial dysfunction-related clusters. The criteria for significant enrichment comprised nominal *p* < 0.05, normalized enrichment score (NES) > 1, and false discovery rate (FDR) q < 0.25 using the GSVA R package.

### 2.8 Analysis of immune cell infiltration

The degree of immune cell infiltration was assessed twice, using 1) CIBERSORT and 2) ssGSEA. First, the CIBERSORT R package was used to determine the degree of immune cell infiltration based on the CIBERSORT scores for immune infiltrating cells (Steen et al., 2020). Second, the GSVA R package method based on ssGSEA (Huang et al., 2021) was used to evaluate the degree of immune cell infiltration.

### 2.9 Statistical analysis

All data processing and analysis were completed in R software (version 4.0.2). Normally distributed continuous variables were compared using independent-samples Student’s t tests, and non-normally distributed continuous variables were compared using Mann–Whitney U tests (i.e., Wilcoxon rank-sum tests). Categorical variables were compared using chi-square tests or Fisher’s exact tests. Kruskal–Wallis tests were used for comparison of more than two groups. Two-tailed *p* < 0.05 was considered statistically significant.

## 3 Results

**FIGURE 1:**
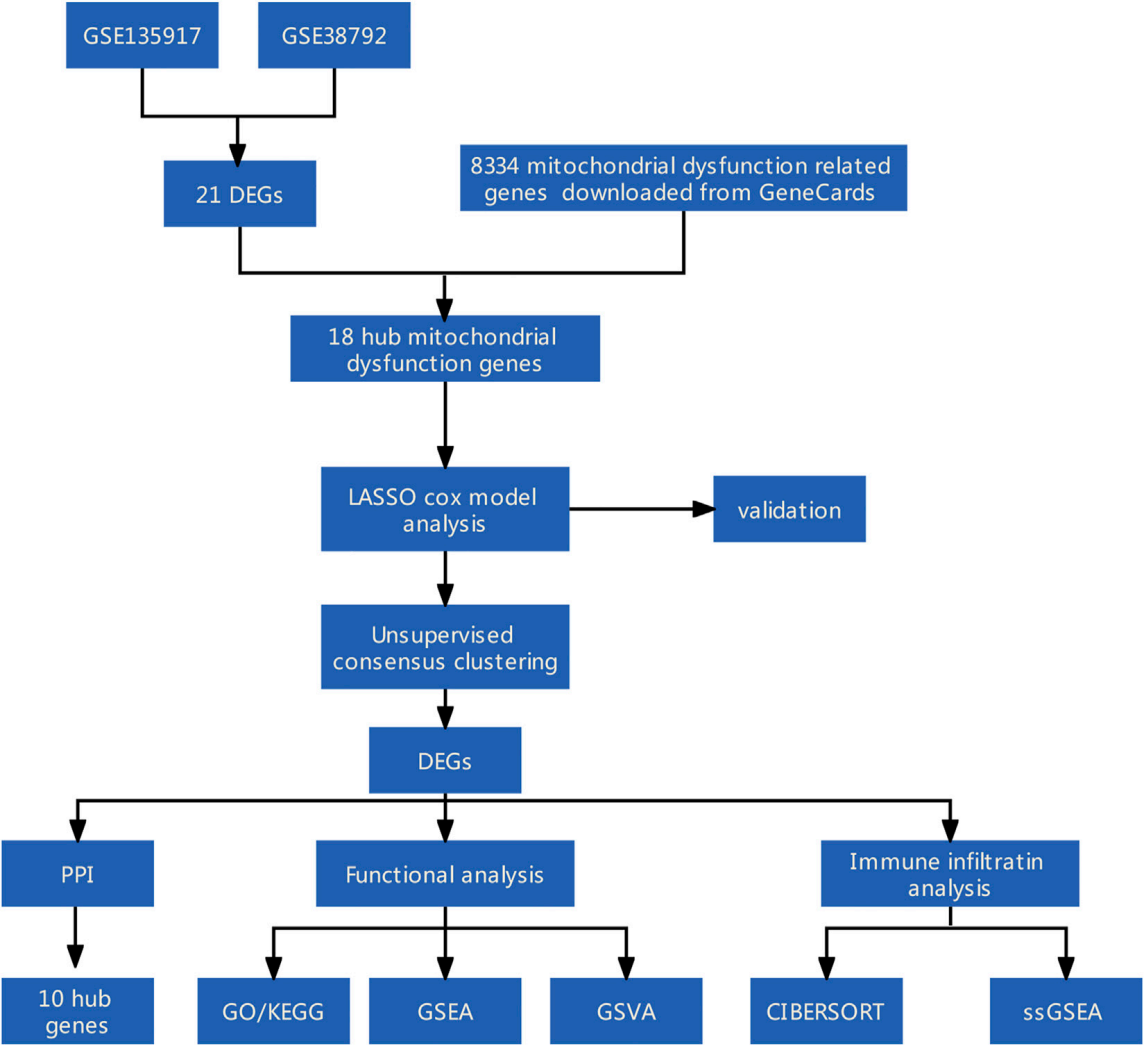
Flow diagram of methodologies used in this study.

Figure 1 shows the study flowchart.

### 3.1 Identification of mitochondrial dysfunction-related differentially expressed genes

**FIGURE 2:**
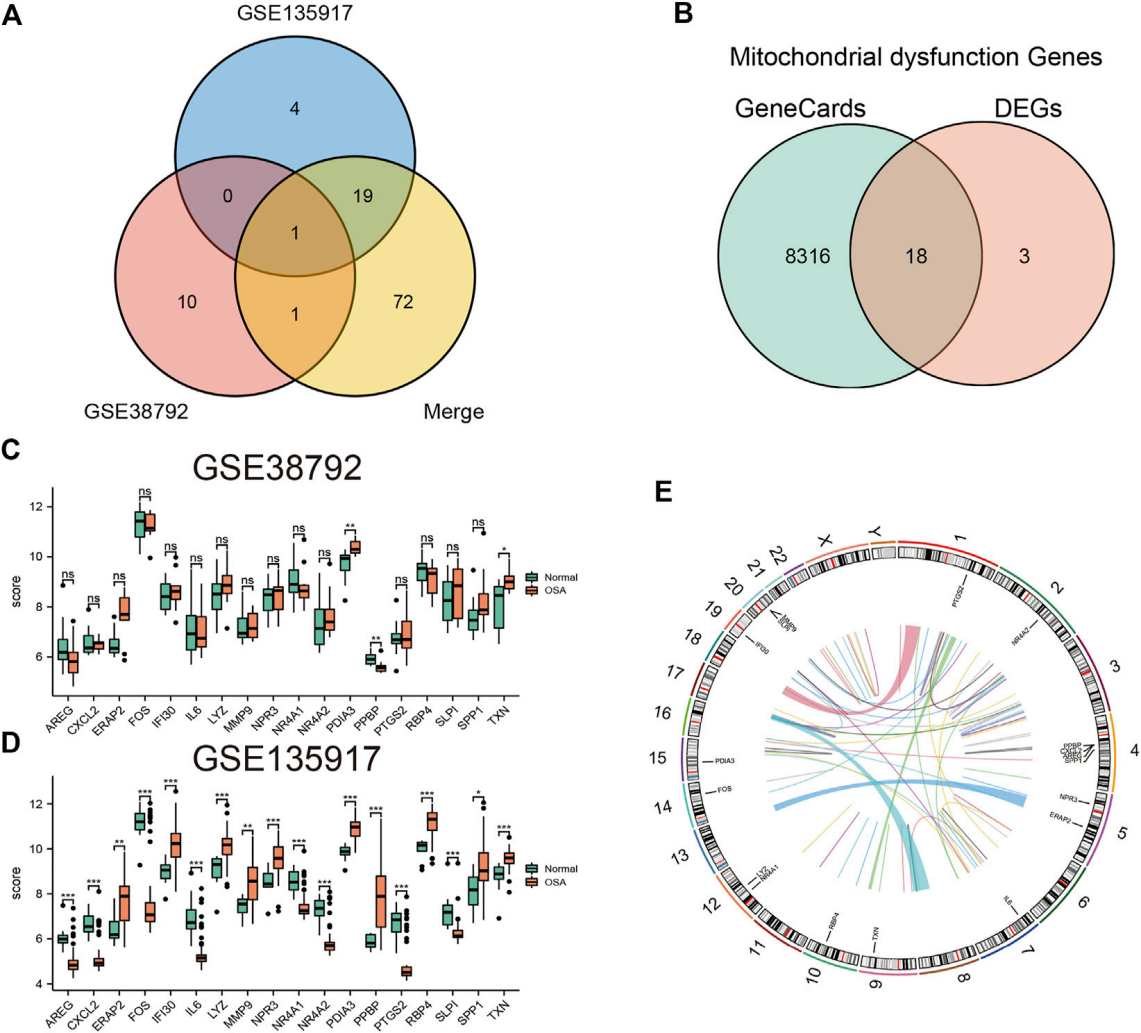
Identification of mitochondrial dysfunction-related hub genes. **(A)** Venn diagram of DEGs between the OSA and control samples in the GSE135917, GSE38792, and combined datasets. **(B)** Venn diagram of hub DEGs and mitochondrial dysfunction-related genes. Boxplots of the differences in expression of mitochondrial dysfunction-related hub genes in **(C)** GSE38792 and **(D)** GSE135917 datasets. **(E)** Chromosomal positions and expression of mitochondrial dysfunction-related hub genes. *p < 0.05, **p < 0.01, ***p < 0.001, ns: no significant difference. DEGs: differentially expressed genes.

First, heatmaps and volcano plots were used to visualize the DEGs between the OSA and control samples in the GSE135917 (Supplementary Figures S2A,B), GSE132651 (Supplementary Figures S2C,D), and combined datasets (Supplementary Figures S2E,F). Next, we analyzed the intersection of DEGs among the GSE135917, GSE38792, and combined datasets, as displayed in a Venn diagram (Figure 2A). A total of 21 overlapping genes were obtained. Moreover, 18 of the overlapping genes were mitochondrial dysfunction-related genes (Figure 2B), which were designated as the mitochondrial dysfunction-related hub genes. Their expression levels in the GSE38792 and GSE135917 datasets are presented in boxplots (Figures 2C,D). Figure 2E shows the chromosomal positions of the mitochondrial dysfunction-related hub genes.

### 3.2 Diagnostic model based on mitochondrial dysfunction-related hub genes

**FIGURE 3:**
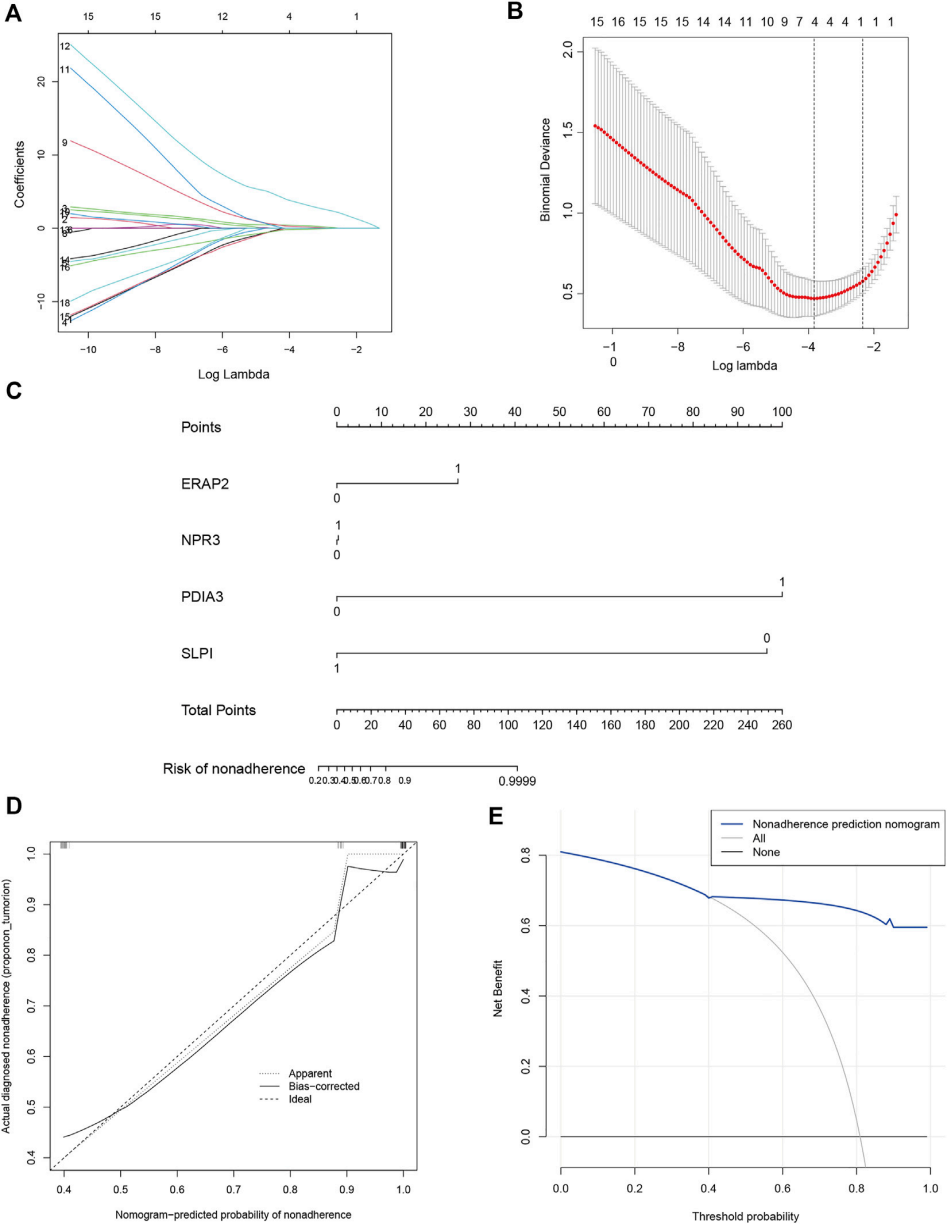
Diagnostic model based on mitochondrial dysfunction-related genes. **(A)** Diagnostic model construction using a least absolute shrinkage and selection operator (LASSO) Cox regression model. **(B)** Coefficient distribution plots to select the optimum lambda value. **(C)** Plot of diagnostic genes demonstrating their differential effectiveness for diagnosing OSA. **(D)** Calibration curves based on nomogram predictions and actual observations. **(E)** Decision curve analysis (DCA) of diagnostic model.

The 18 mitochondrial dysfunction-related hub genes were subjected to LASSO Cox regression to create a diagnostic model (Figure 3A). Four genes were gathered, the regression model reached the optimal ability (Figure 3B). A plot of the diagnostic genes was used to visualize their differential effectiveness for diagnosing OSA (Figure 3C). The calibration curve regarding the nomogram predictions (Figure 3D) and decision curve analysis curve predicted by irrelevant nomogram (Figure 3E) were constructed. Both showed that 4− gene diagnostic model had good predictive value.

### 3.3 Verification of diagnostic value of four-gene diagnostic model

**FIGURE 4:**
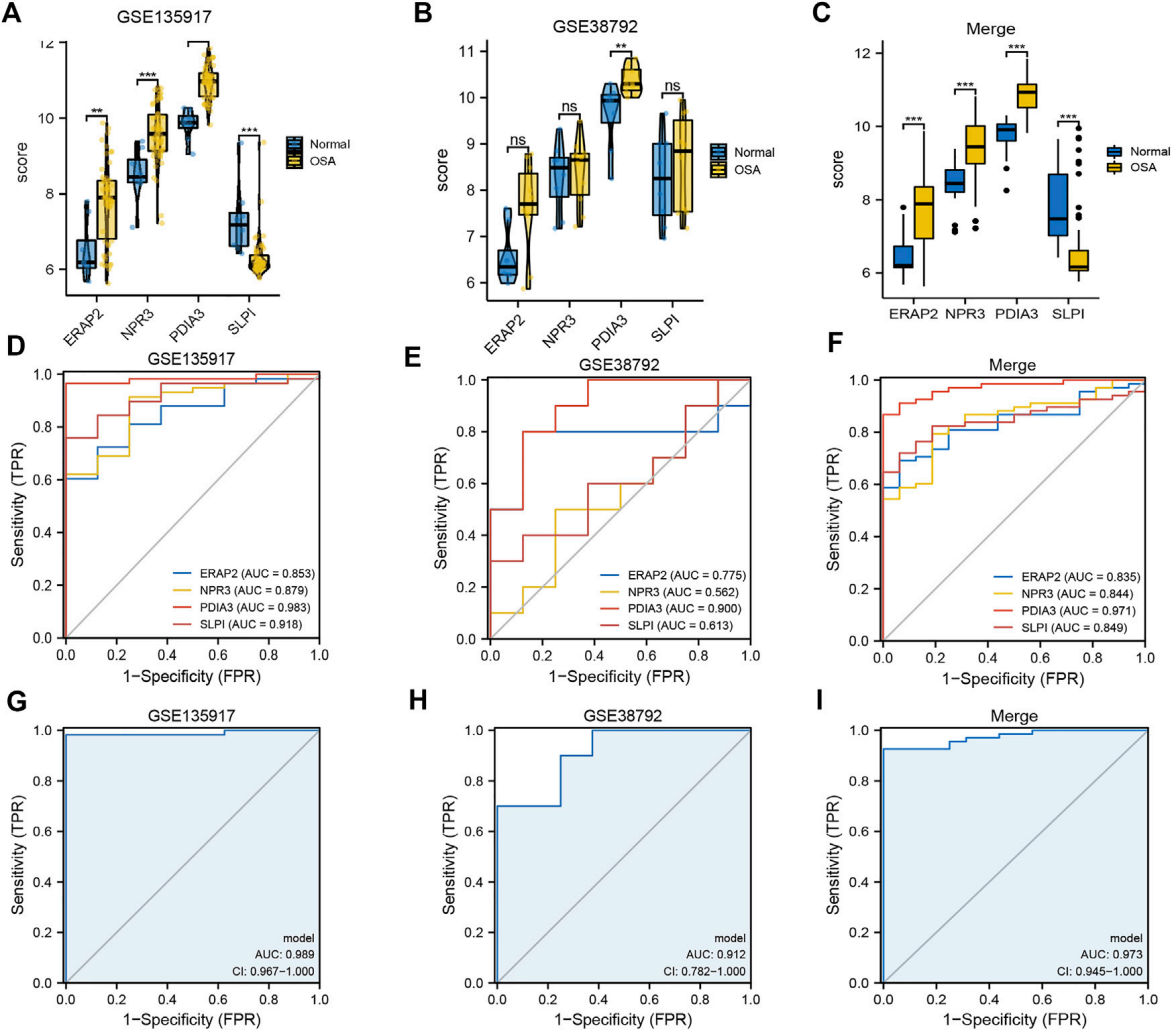
Expression differences and diagnostic value of mitochondrial dysfunction-related four-gene diagnostic model. Boxplots of differences in NPR3, PDIA3, SLPI, and ERAP2 expression between OSA and control samples in **(A)** GSE135917, **(B)** GSE38792, and **(C)** combined datasets. ROCcurves of NPR3, PDIA3, SLPI, and ERAP2 in **(D)** GSE135917, **(E)** GSE38792, and **(F)** combined datasets. ROC curves of four-gene diagnostic model in **(G)** GSE135917, **(H)** GSE38792, and **(I)** combined datasets.

Boxplots of the four genes (*NPR3, PDIA3, SLPI,* and *ERAP2*) in the diagnostic model, as OSA-related risk genes, had significant differences in expression between the OSA and control samples in the GSE135917, GSE38792, and combined datasets (Figures 4A–C).

The area under the ROC curve (AUC) was calculated to measure the diagnostic value of the model. Figures 4D–F show the ROC curves of *NPR3, PDIA3, SLPI, and ERAP2* in the GSE135917, GSE38792, and combined datasets, respectively. Figures 4G–I show the ROC curves of the four-gene diagnostic model in the GSE135917, GSE38792 and combined datasets, respectively. The results indicated that the four-gene signature of diagnostic model had high diagnostic value.

### 3.4 Mitochondrial dysfunction-related clusters

**FIGURE 5:**
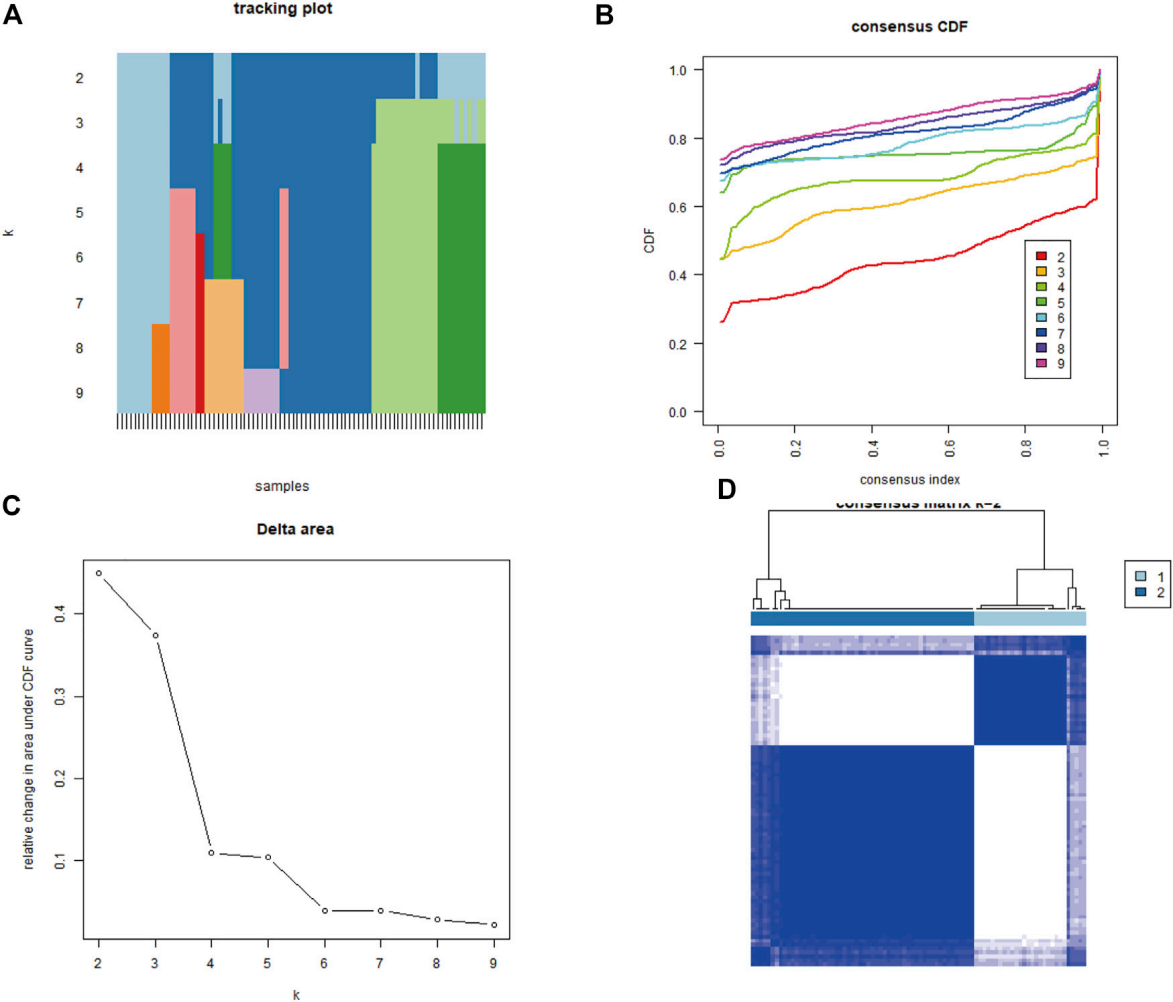
Identification of two mitochondrial dysfunction-related clusters using consensus clustering analysis of mitochondrial dysfunction-related genes. **(A)** Tracking plot of consistent clustering. **(B)** Cumulative distribution function (CDF) curves for k = 2–9. **(C)** Elbow plot showing relative change in area under the CDF curve (AUC). **(D)** Consensus clustering matrix for k = 2.

To explore biological characteristics related to the expression of mitochondrial dysfunction-related genes, all the samples were first divided into k (k = 2, 3, 4, 5, 6, 7, and 8) clusters using ConsensusClusterPlus. The optimal categorization occurred when k = 2, based on the cumulative distribution function (CDF) curves of the consensus score. Therefore, the samples were divided into two mitochondrial dysfunction-related clusters: cluster A (*n* = 28) and cluster B (*n* = 56) (Figures 5A–D).

### 3.5 Transcription factor and miRNA predictions

To explore the interactions related to four-gene diagnostic model at the post-transcriptional level, 41 transcription factors that upregulate the genes and 134 miRNAs that target the genes were identified (Supplementary Figure S3A), along with 104 protein chemical components (Supplementary Figure S3B).

### 3.6 Protein–protein interaction network

**FIGURE 6:**
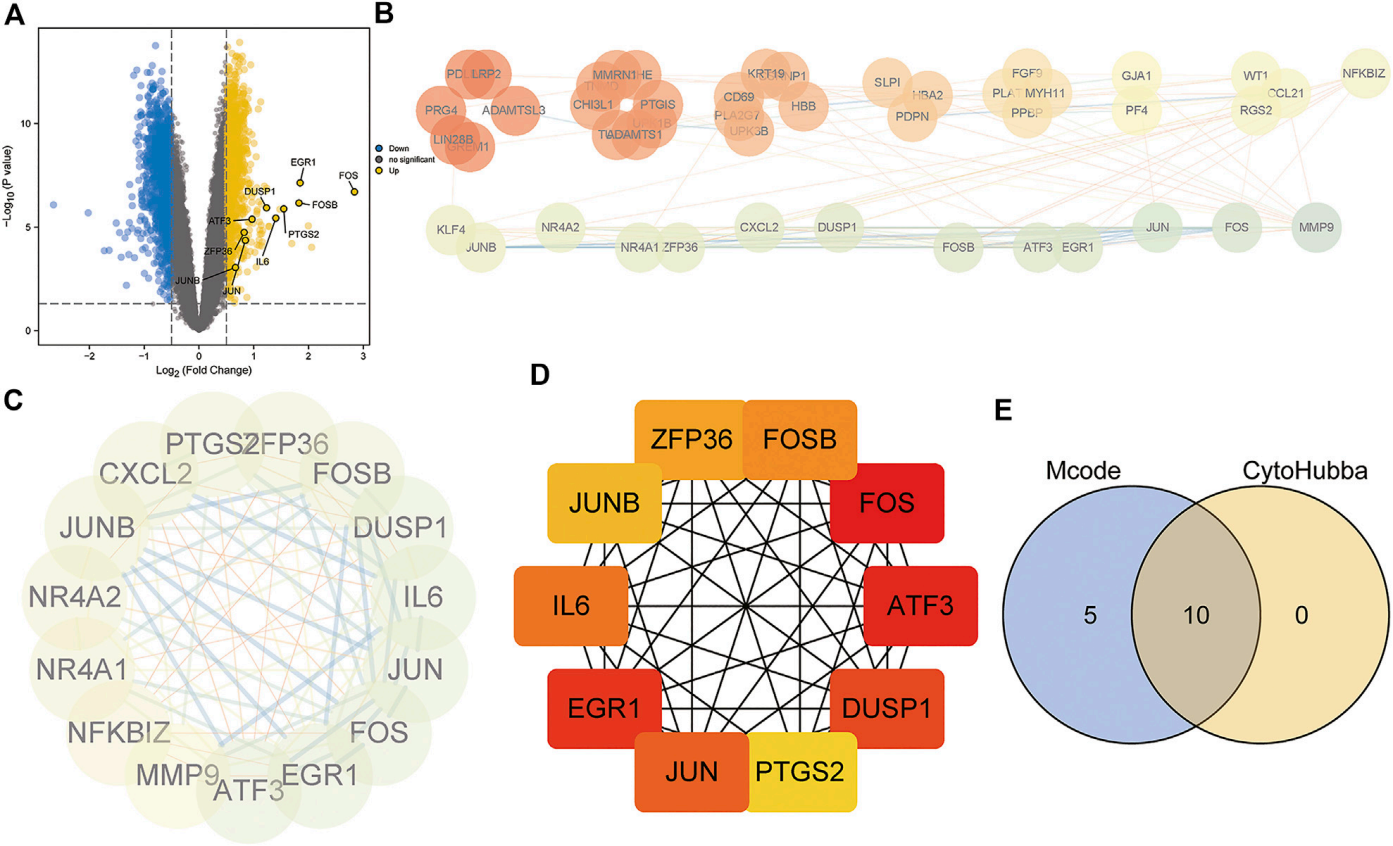
Protein–protein interaction (PPI) network construction and hub gene identification. **(A)** Volcano plot of DEGs between mitochondrial dysfunction-related clusters A and B. **(B)** PPI network based on STRING database. **(C)** Hub genes identified by Cytoscape MCODE plug-in. **(D)** Top10 hub genes identified by Cytoscape CytoHubba plug-in. **(E)** Venn diagram showing the intersection of the two methods, identifying 10 hub genes.

To investigate the underlying biological functions of the mitochondrial dysfunction-related clusters A and B, we identified 106 DEGs between clusters A and B (Figure 6A). We then constructed a PPI network using the STRING database (Figure 6B). The highly connected (hub) genes in the PPI network were identified using the MCODE plug-in in Cytoscape (Figure 6C). The top 10 genes, based on high scores using the cytoHubba plug-in in Cytoscape, were also selected (Figure 6D). A Venn diagram was used to identify the intersection of the results of the MCODE and cytoHubba methods, which led to 10 hub genes being obtained (Figure 6E). A correlation network diagram (Supplementary Figure S4A), scatter plots (Supplementary Figures S4B–P) and Supplementary Table S2 show the correlations of the 10 hub genes and 4 genes in the diagnostic model, revealing strong correlations between the hub genes and diagnostic genes. This indicated that they may act in synergistic way, contributing to OSA and related complications.

### 3.7 Functional enrichment analyses

**FIGURE 7:**
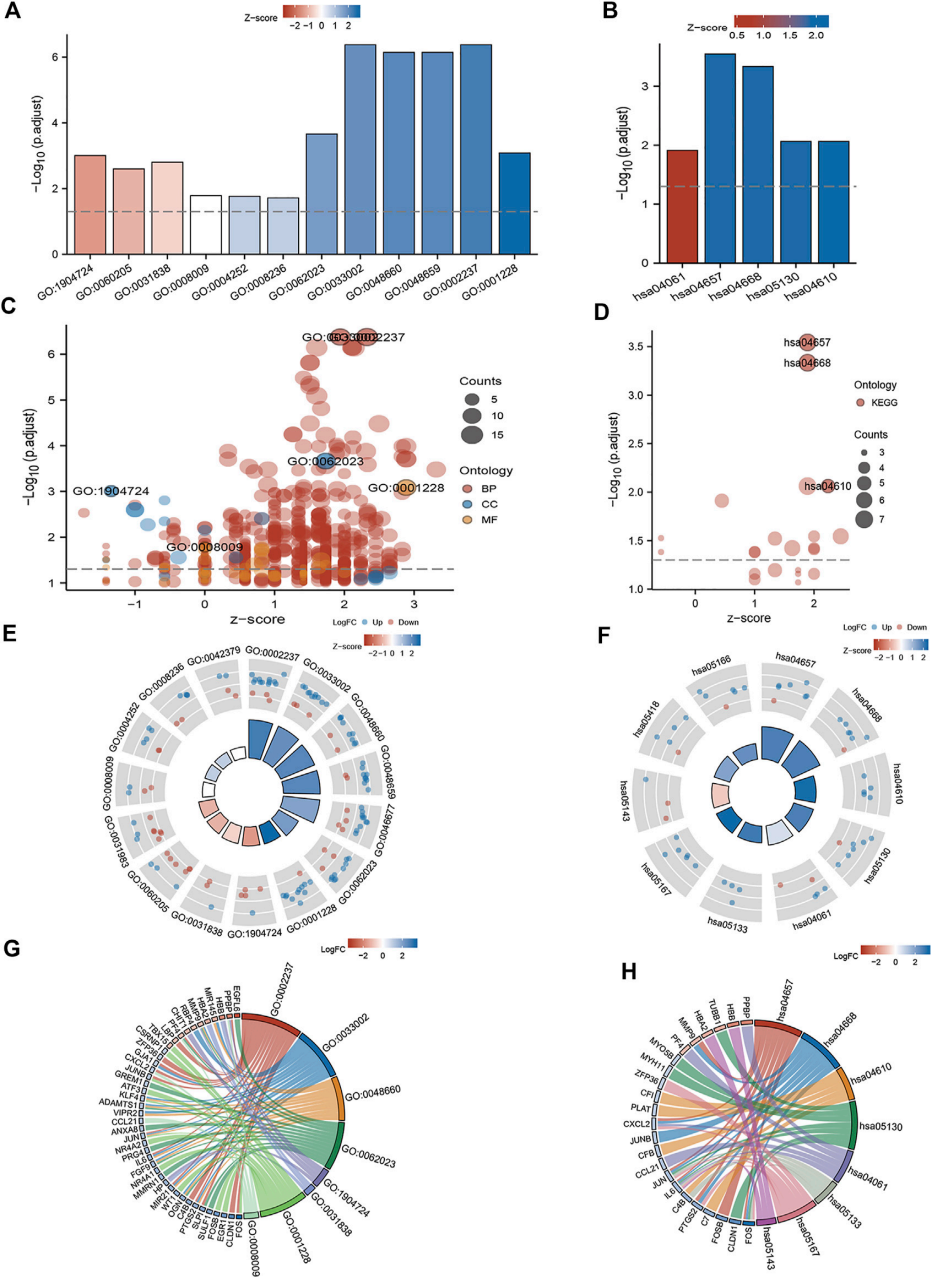
GO and KEGG enrichment analyses of DEGs between mitochondrial dysfunction-related clusters A and B. **(A,B)** Histogram, **(C,D)** bubble plot, **(E,F)** circle plot, and **(G,H)** chord diagram of the results of GO and KEGG enrichment analyses.

Functional enrichment analyses of the 106 DEGs between clusters A and B were performed (Figure 7). The GO analysis indicated that the genes were significantly enriched in cytoplasmic vesicle lumen, chemokine activity, collagen-containing extracellular matrix, regulation of smooth muscle cell proliferation, DNA binding, and transcription activator activity (Supplementary Table S3). The KEGG analysis showed that the genes were enriched in cytokine and cytokine receptor, interleukin (IL)-17 signaling pathway, tumor necrosis factor (TNF) signaling pathway, pathogenic *Escherichia coli* infection, and complement and coagulation cascades (Supplementary Table S4).

**FIGURE 8:**
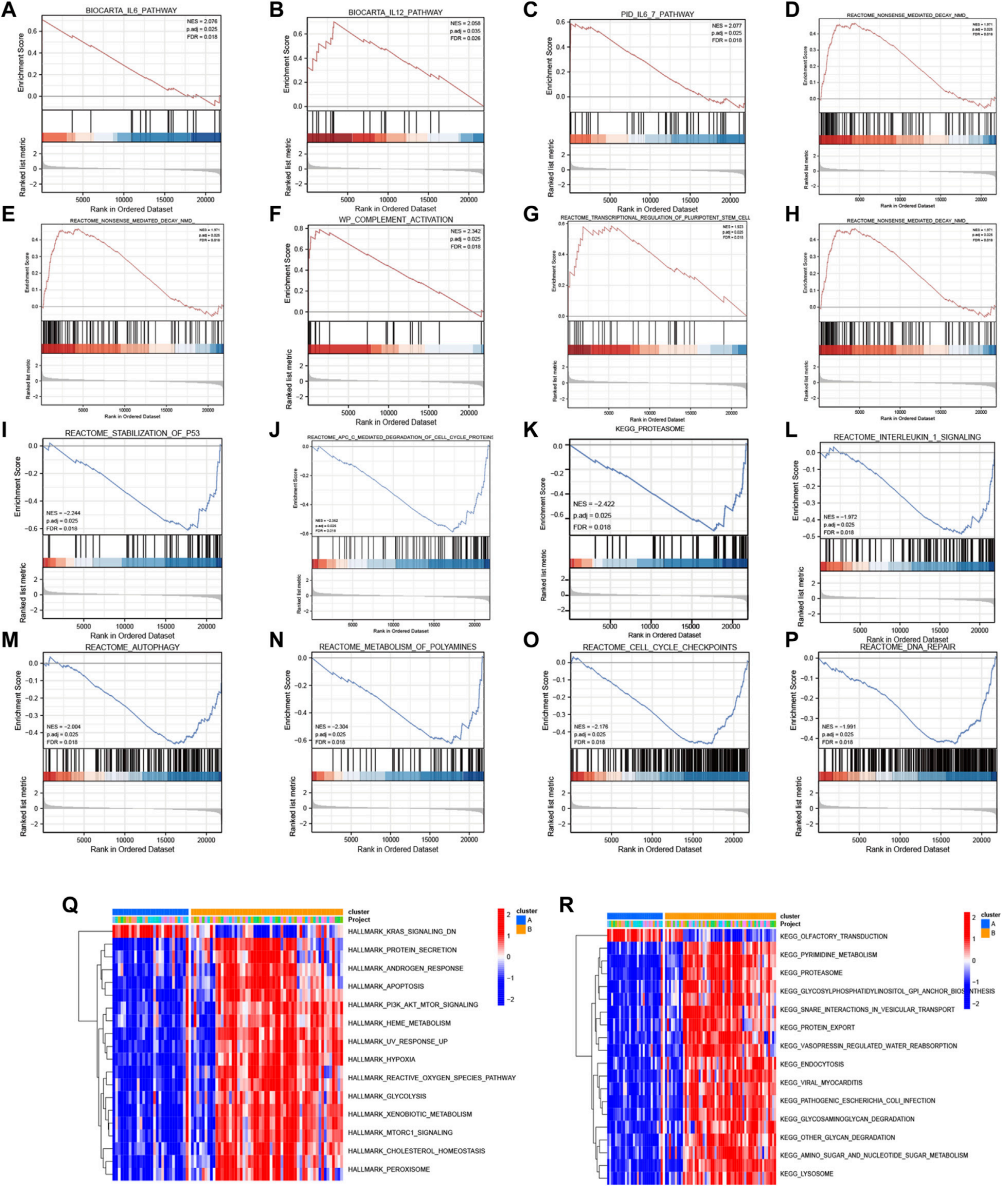
GSEA and GSVA. GSEA results: KEGG pathways with significantly differential enrichment between patients with **(A–H)** high and **(I–P)** low expression of the four diagnostic genes. GSVA results: **(Q)** Hallmark and **(R)** KEGG pathways with significantly differential enrichment between mitochondrial dysfunction-related clusters A and B.

Subsequently, GSEA was performed between the high- and low-expression clusters based on the four diagnostic genes in the GSE135917 and GSE38792 datasets (Supplementary Table S5). The results suggested that the samples in the high-expression cluster were significantly enriched in IL-6 pathway, IL-12 pathway, IL6_7 pathway, DNA repair, IL-1 signaling, nonsense-mediated decay, transcriptional regulation of pluripotent stem cells, complement activation, and gastrin signaling pathway (Figures 8A–H). The samples in the low-expression cluster were significantly enriched in autophagy, lysosome, proteasome, anaphase promoting complex/cyclosome (APC/C)-mediated degradation of cell cycle proteins, cell cycle check points, metabolism of polyamines, and stabilization of P53 (Figures 8I–P). Cytokines exert a vast array of immunoregulatory actions critical to human physiology and disease (Spangler et al., 2015). TNF-α, IL-17, IL-6, IL-12, and IL-1 are inflammatory cytokines. The autophagy–lysosome pathway and ubiquitin–proteasome system are the main mechanisms of intracellular protein degradation and they help to maintain normal cellular functions.

To further investigate the biological pathways that mitochondrial dysfunction may affect, we conducted GSVA between the mitochondrial dysfunction-related clusters A and B to assess pathway enrichment. Regarding the KEGG pathways, most of them, including regulation of pyrimidine metabolism, proteasome, SNARE interactions in vesicular transport, endocytosis, other glycan degradation, amino sugar and nucleotide sugar metabolism, and lysosome, were more enriched in cluster B (Figure 8Q). Regarding the Hallmark pathways, the ROS pathway, heme metabolism, PI3K-AKT-mTOR signaling, mTORC1 signaling, hypoxia, peroxisome, and apoptosis were more enriched in cluster B, whereas myogenesis and KRAS signaling pathways were more enriched in cluster A (Figure 8R). ROS influence metabolic processes such as proteasome function, autophagy, and general inflammatory signaling (Forrester et al., 2018). Heme metabolism influences a wide variety of biological processes relevant to OSA, including redox balance and inflammatory response (Wang et al., 2022). Autophagy, which can be activated by hypoxia, can be beneficial in inflammatory disorders as it eliminates damaged organelles and maintains homeostasis (Yao et al., 2021), and mTOR is a key negative regulator of autophagy. It should be noted that the enriched pathways were mainly autophagy, inflammatory, and immune related pathways. This indicated that mitochondrial dysfunction might be associated with autophagy, inflammation, and the immune microenvironment in OSA.

### 3.8 Immune cell infiltration

**FIGURE 9:**
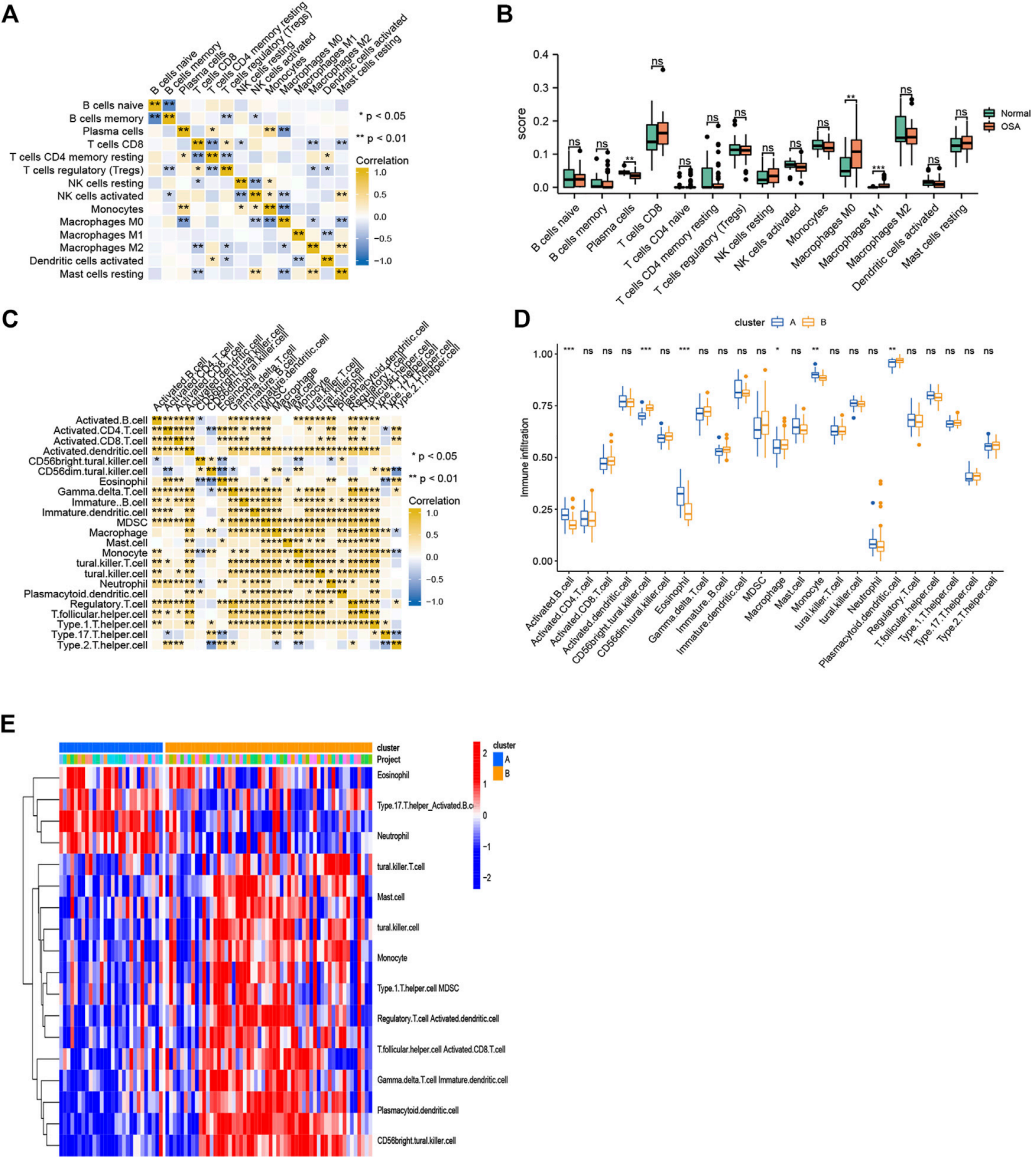
Immune cell infiltration. **(A)** Heatmap of correlations among 15 infiltrating immune cells, as analyzed by CIBERSORT. **(B)** Boxplot of differences in 15 infiltrating immune cells between OSA and control samples, as analyzed by CIBERSORT. **(C)** Heatmap of correlations among 23 infiltrating immune cells, as analyzed by ssGSEA. **(D)** Boxplot of differences in 23 infiltrating immune cells between clusters A and B, as analyzed by ssGSEA. **(E)** Heatmap of the differences in immune cell infiltration (based on ssGSEA between clusters A and B.

The CIBERSORT algorithm was used to evaluate the immune microenvironment in OSA. The correlations among immune cells are shown in Figure 9A. A boxplot indicated that the infiltration of several immune cells (plasma cells, M0 macrophages, and M1 macrophages) were significantly different between OSA and control samples (Figure 9B).

To explore the relationship between mitochondrial dysfunction and immune cell infiltration, we compared the immune cell infiltration between clusters A and B. The results showed that several immune cells (activated B cells, CD56^bright^ natural killer cells, eosinophils, macrophages, monocytes, and plasmacytoid dendritic cells) were significantly different between clusters A and B (Figures 9C,D). Figure 9E further visualizes immune cell infiltration differences between clusters.

**FIGURE 10:**
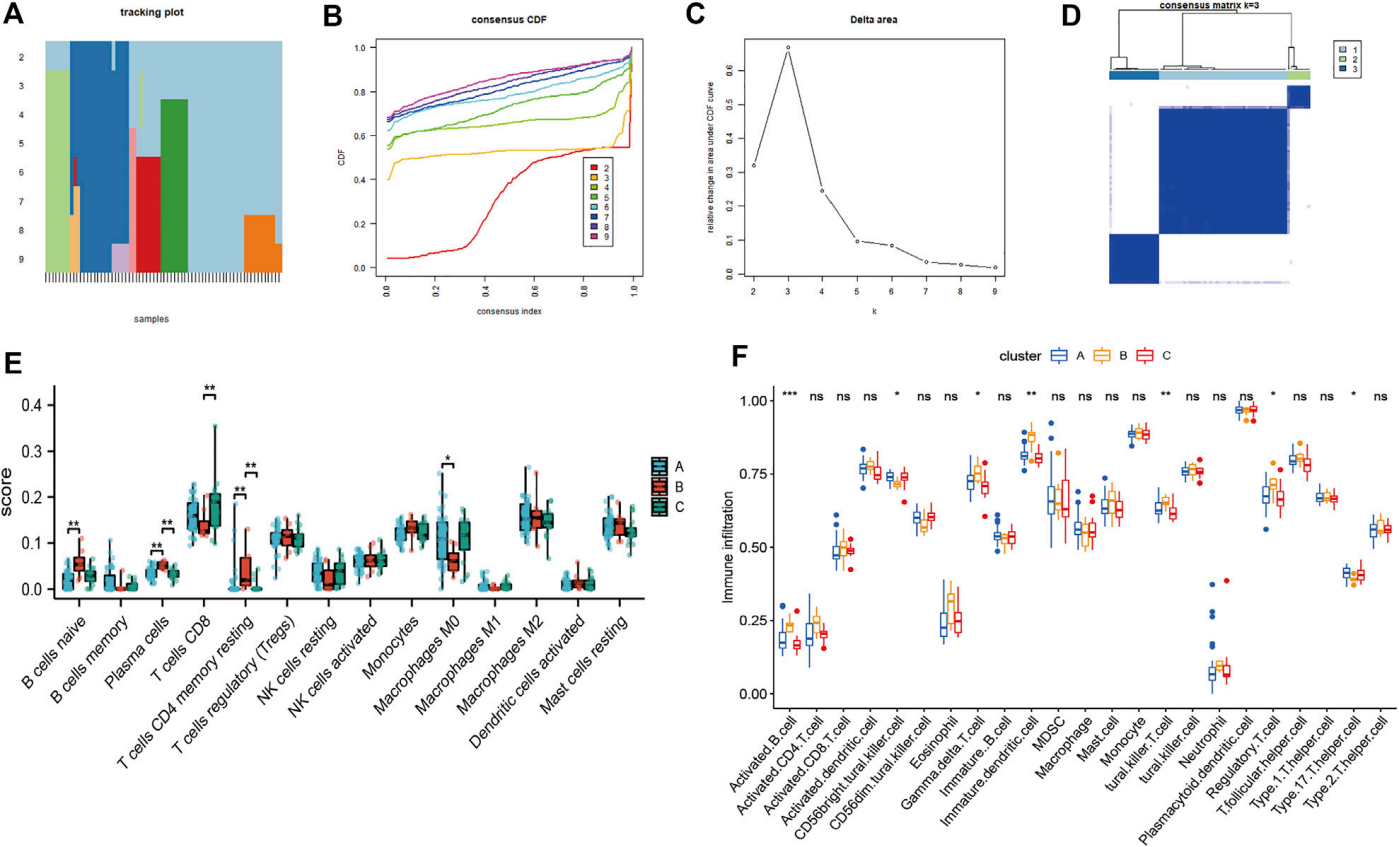
Consensus clustering analysis of OSA subjects based on mitochondrial dysfunction-related genes and analysis of immune cell infiltration. **(A)** Tracking plot of consistent clustering. **(B)** Consensus clustering cumulative distribution function (CDF) for k = 2–9. **(C)** Relative change in area under CDF curve for k = 2–7. **(D)** OSA subjects were divided into three clusters when k = 3. Boxplots of degree of immune cell infiltration among clusters A–C, based on **(E)** CIBERSORT and **(F)** ssGSEA methods.

We then performed consensus clustering of the OSA samples only (*n* = 68) and identified three mitochondrial dysfunction-related clusters, with 43 samples in cluster A, 8 in cluster B, and 17 in cluster C (Figures 10A–D). We assessed the degree of immune cell infiltration using both CIBERSORT and ssGSEA. Interestingly, the results were consistent with each other. Both methods showed differences among clusters A, B, and C in the infiltration degree of activated B cells, CD56^bright^ natural killer cells, γ/δ T cells, immature dendritic cells, natural killer T cells, regulatory T cells (Tregs), and type 17 T helper cells (Figures 10E,F).

To understand the correlations between the genes in the diagnostic model and infiltrating immune cells, we constructed a scatter plot of statistically significant results with correlation coefficient (R) > 0.4. *PDIA3* was correlated with plasma cells, monocytes, M0 macrophages and T cells CD4 memory resting (Supplementary Figures S5A–D). *SLPI* was correlated with M0 macrophages, naive B cells, and plasma cells (Supplementary Figures S5E–G). Taken together, the results indicate that mitochondrial dysfunction plays an important role in immune microenvironment regulation in OSA.

## 4 Discussion

OSA can cause many complications, such as cardiovascular, metabolic, and neuropsychiatric disorders, pose a major threat to human health (Wang et al., 2022). However, the approaches to the management of OSA are limited due to the incomplete understanding of the underlying molecular mechanisms of OSA.

During respiratory events in OSA patients, intermittent hypoxia together with post-apnea/hypopnea reoxygenation triggers an increase in oxidative stress (Passali et al., 2015). As the major energy-producing organelles, mitochondria, are highly sensitive to hypoxic stress. They can respond dynamically under hypoxia, which can minimize ROS formation and reduce the risk of cell death and tissue damage. However, as a prominent mechanism of mitochondrial dysfunction, abnormal metabolic cues induced by hypoxia can disrupt the dynamic mitochondrial balance. This results in a series of intracellular signaling cascades and apoptosis, followed by the progression of diverse diseases (Wang et al., 2022). Mitochondrial abnormalities may be one of the pathological mechanisms underlying OSA-related cardiac injury, while maintaining the integrity of mitochondria allows the survival of cardiomyocytes under hypoxia. Aged relative to young mouse hearts exhibited maladaptation to CIH because of mitochondrial dysfunction (Wei et al., 2022).

Since the mitochondrial dysfunction appears to be involved in the pathogenesis of OSA and its complications, investigating the role of mitochondrial dysfunction-related genes may provide novel personalized and optimal management strategies for OSA and its comorbidities. In the present study, we identified a mitochondrial dysfunction-related four-gene signature of diagnostic model involving *NPR3, PDIA3, SLPIM,* and *ERAP2*. The model easily distinguished between OSA and control samples, which highlights that mitochondrial dysfunction differs between OSA patients and control individuals. Although there have been previous studies on OSA diagnostic genes (Li et al., 2017; Ambati et al., 2020; Bencharit et al., 2021; Cao et al., 2021; Li et al., 2022), we are the first group to establish and validate a mitochondrial dysfunction-related diagnostic model.

Among the mitochondrial dysfunction-related genes, *NPR3* mediates natriuretic peptides degradation and was proved to act as a tumor suppressor in certain types of cancers. Moreover, previous study also showed that it played an important role in modulating intravascular volume and vascular tone and could protect cardiomyocytes from apoptosis. Thus, *NPR3* might be a viable therapeutic target to decrease cancer and cardiovascular diseases risk in OSA patients (Lin et al., 2016; Li et al., 2021). *PDIA* was reported to be able to intercept the endoplasmic reticulum stress-related apoptotic cellular death and its expression is significantly up-regulated in response to cellular stress (Mahmood et al., 2021). *SLPI* is an important immunity regulator, acts as a component of tissue regenerative programs, and has anti-proteolytic, anti-microbial and immunomodulatory activities (Majchrzak-Gorecka et al., 2016). *ERAP2* plays roles in the processing of antigenic peptides and influences cellular cytotoxic immune responses (de Castro and Stratikos 2019). Obviously, *PDIA3*, *SLPI,* and *ERAP2* are involved in stress and immune response. The experimental models of OSA suggested that the metabolic and inflammatory changes induced by chronic intermittent hypoxia and sleep fragmentation may foster or exacerbate immune alterations (Almendros et al., 2020).

We obtained 134 miRNAs related to the four genes in the mitochondrial dysfunction-related diagnostic model. A previous study reported differentially expressed miRNAs in OSA (Li et al., 2017), but they were not necessarily associated with mitochondrial dysfunction. Additionally, to identify another set of key genes related to OSA, we selected 10 hub genes (*IL-6, FOS, FOSB, JUN, DUSP1, EGR1, PTGS2, ATF3,* and *ZFP36*) from the PPI network of DEGs between the two mitochondrial dysfunction-related clusters. Interestingly, IL-6 receptor levels have been reported to reflect OSA severity (Zheng et al., 2018); *FOS, FOSB,* and *JUN* have been demonstrated to be involved in obesity, osteoporosis, and colorectal cancer (Skrypnik et al., 2017); and *DUSP1* is upregulated in CIH in OSA patients (Hoffmann et al., 2013). Additionally, in this study, we found that *SLP1* expression was positively correlated with *IL-6, FOS, FOSB,* and *JUN*, whereas *PDIA3* expression was negatively correlated with *FOS, FOSB,* and *JUN*. Although most of the 10 hub genes have not been studied in OSA, we speculate that these genes might be involved in the pathogenesis of OSA and its complications and form a regulatory network to coregulate OSA.

Functional enrichment analysis was conducted after reclassifying the microarray according to the mitochondrial dysfunction and our results indicated that DEGs of two clusters were primarily involved in autophagy, inflammation and immune pathways. 1) Regarding autophagy, consistent with our results, OSA is known to induce autophagy as a result of hypoxia, oxidative stress, and endothelial dysfunction (Ding et al., 2021). Autophagy is related to metabolic disorders, tumors, pulmonary diseases, and neurodegenerative disorders, and mitophagy is an autophagic response that specifically targets mitochondria (Bravo-San Pedro et al., 2017). 2) Regarding inflammation, mitochondrial dysfunction can trigger innate immune responses and inflammation (West 2017). Additionally, inflammatory mediators and infiltrating immune cells can trigger signaling cascades that alter mitochondrial metabolism. Cytokines can inhibit mitochondrial oxidative phosphorylation and induce mitochondrial ROS production, which may alter mitochondrial dynamics and ultimately result in cell death. In particular, it has been reported that OSA may lead to atherosclerosis due to inflammatory processes induced by CIH (Stanke-Labesque et al., 2014). 3) Regarding immune pathways, high levels of IL-6 and TNF-α are predictors of major adverse cardiovascular events in diabetic patients with peripheral artery disease (Biscetti et al., 2019). The IL-1 superfamily of cytokines also plays a vital role in immunity by regulating host defenses, inflammation, and injury. IL-1 inhibition improves glycemic control, and decreases the incidence of cardiovascular disease (Herder et al., 2015; Zheng et al., 2019). Notably, IL-33, a cytokine from the IL-1 family, is an inflammatory mediator, that is, increased in OSA patients compared to controls (Gabryelska et al., 2019). Importantly, pro-inflammatory activation of monocytes activates mTORC1, which enhances the production of chemokines and cytokines (Lin et al., 2014), and the mTORC1 pathway was found to play a key role in mitochondrial dysfunction (Condon et al., 2021). The mTOR pathway was also reported to be the most important DEG-enriched pathway in severe OSA patients with hypertension (Ko et al., 2021). In summary, these results gave a detailed description of the ways and mechanisms how mitochondrial dysfunction participates in OSA’s progress, which may benefit future development of precise treatment.

Our data demonstrated the differences in infiltrating immune cells between OSA and control samples, and these cells may also be responsible for OSA comorbidities. Immune cell infiltration may also be of great importance in the remission of OSA (Fan et al., 2021). Consistent with our findings, CIH in OSA was previously found to induce adipose tissue macrophages towards a pro-inflammatory M1 subtype (Ryan 2017), and macrophages are known to contribute to adipose tissue insulin resistance and vascular atherogenesis (Trzepizur et al., 2018). Additionally, imbalanced effector T helper cells were found in patients with OSA and hypertension (Zhang et al., 2022). Moreover, immune cell infiltration in the myocardium adversely affects heart function (Carrillo-Salinas et al., 2019), so OSA may elicit the immunologic alterations that lead to cardiovascular changes. Lastly, there is also a link between OSA and increased cancer incidence and mortality, as intermittent hypoxia induces changes in signaling pathways involved in the regulation of host immunological surveillance that results in tumor formation and invasion (Martínez-García et al., 2016; Picado and Roca-Ferrer 2020). Intermittent hypoxia may lead to a tumor-promoting phenotype among tumor-associated macrophages, leading to more aggressive tumor behavior (Cao et al., 2015). Better understanding of immune infiltration may be of great significance discovering novel therapeutic targets and improving cardiovascular and cancer outcomes in OSA.

Mitochondria are not only important for energy supply during immune activation, but they also induce host immunological surveillance and are involved in immune cell differentiation. We found that several immune cell types, especially T cells (γ/δ T cells, natural killer T cells, Tregs, and type 17 T helper cells), were significantly different among the three mitochondrial dysfunction-related clusters of OSA samples, so T cells are a promising choice for future OSA treatment development. In addition, the diagnostic genes were correlated with immune cell infiltration. Research has shown that mitochondrial processes, along with cytosolic metabolic processes, drive T cell activation, survival, proliferation, and effector functions (Sena et al., 2013). Another study showed that increased γ/δ T cell adhesion occurs in lesion-prone areas of the arterial tree when high cholesterol induces the translocation of ATP synthase *β* chain from the mitochondria to membrane caveolae in endothelial cells (Fu et al., 2011). Furthermore, research showed that mTORC1 activation induces Treg proliferation while inhibiting the suppressive activity of the Tregs (Procaccini et al., 2010), which suppress immune activation *via* immunosuppressive cytokines (Agita and Alsagaff 2017). Lastly, T cells have been shown to be central to the immune responses contributing to hypertension (Madhur et al., 2021). In summary, our research revealed that mitochondrial dysfunction might influence immune cell infiltration, including T cell infiltration, in OSA and thus promote OSA-related diseases.

However, our study has several limitations. First, the sample size was small. To confirm the diagnostic value for OSA of the hub genes, external validation using a larger sample size would be helpful. Second, not only the identification of mRNA expression level by real time PCR, but also the protein expression levels of these genes would be necessary to examine using western blot to deepen our understanding the molecular mechanisms of OSA. To comprehensively identify the nature of the mitochondrial dysfunctions in OSA, integrated analysis at the molecular, cellular, and organismal levels is warranted, with experimental evidence being required to fully determine the roles of the hub genes and the underlying mechanisms of OSA. Third, the role of each hub gene and the mechanisms underlying OSA were not fully elucidated. Further experimental verifications are necessary to elucidate the biological functions of these genes in OSA. Fourthly, the correlations between the expression of these genes and the clinical parameters of OSA were not explored, so further research on this is required.

In conclusion, we established and validated a mitochondrial dysfunction-related four-gene signature of diagnostic model for OSA. Moreover, we revealed that this model was related to immune cell infiltration. The model could act as a diagnostic biomarker model and might provide therapeutic targets the treatment of OSA. Further studies should be conducted to clarify our findings.

## Data Availability

The datasets presented in this study can be found in online repositories. The names of the repository/repositories and accession number(s) can be found in the article/Supplementary Material.

## Supplementary material

Supplementary Figure S1 Normalization of datasets. Gene expression distribution in **(A)** GSE38792 dataset before normalization, **(B)** GSE38792 dataset after normalization, **(C)** GSE135917 dataset before normalization, **(D)** GSE135917 dataset after normalization, **(E)** combined dataset before normalization, and **(F)** combined dataset after normalization.

Supplementary Figure S2 Differentially expressed genes (DEGs) between OSA and control samples. Heatmaps and volcano plots of DEGs between OSA and control samples in **(A,B)** GSE38792, **(C,D)** GSE135917, and **(E,F)** combined datasets. The upregulated, downregulated, and non-significant genes are marked in blue, yellow and grey dots, respectively.

Supplementary Figure S3
**(A)** Prediction of transcription factors and miRNAs related to the genes in the four-gene diagnostic model. Red and green dots in the middle represent genes related to mitochondrial dysfunction and their related transcription factors, respectively. Blue dots in the outer layer represent the related miRNAs. **(B)** Molecular networks of protein chemical interactions of the genes in the four-gene diagnostic model.

Supplementary Figure S4 Correlations of 10 hub genes and 4 diagnostic genes. **(A)** Correlation network diagram of correlations among hub gene expression levels. Scatter plots of significant **(B–H)** negative and **(I–P)** positive correlations between hub and diagnostic gene expression.

Supplementary Figure S5 Correlations between genes in the diagnostic model and infiltrating immune cells in OSA samples. Scatter plots of significant correlations of **(A–E)** SLPI and **(F–H)** PDIA3 expression with the degree of immune cell infiltration.
