# Supplementary figures and images for: Construction of a mitochondrial dysfunction related signature of diagnosed model to obstructive sleep apnea
Source: Front Genet. 2022 Nov 21;13:1056691. doi: 10.3389/fgene.2022.1056691 (PMC9714559; doi:10.3389/fgene.2022.1056691)

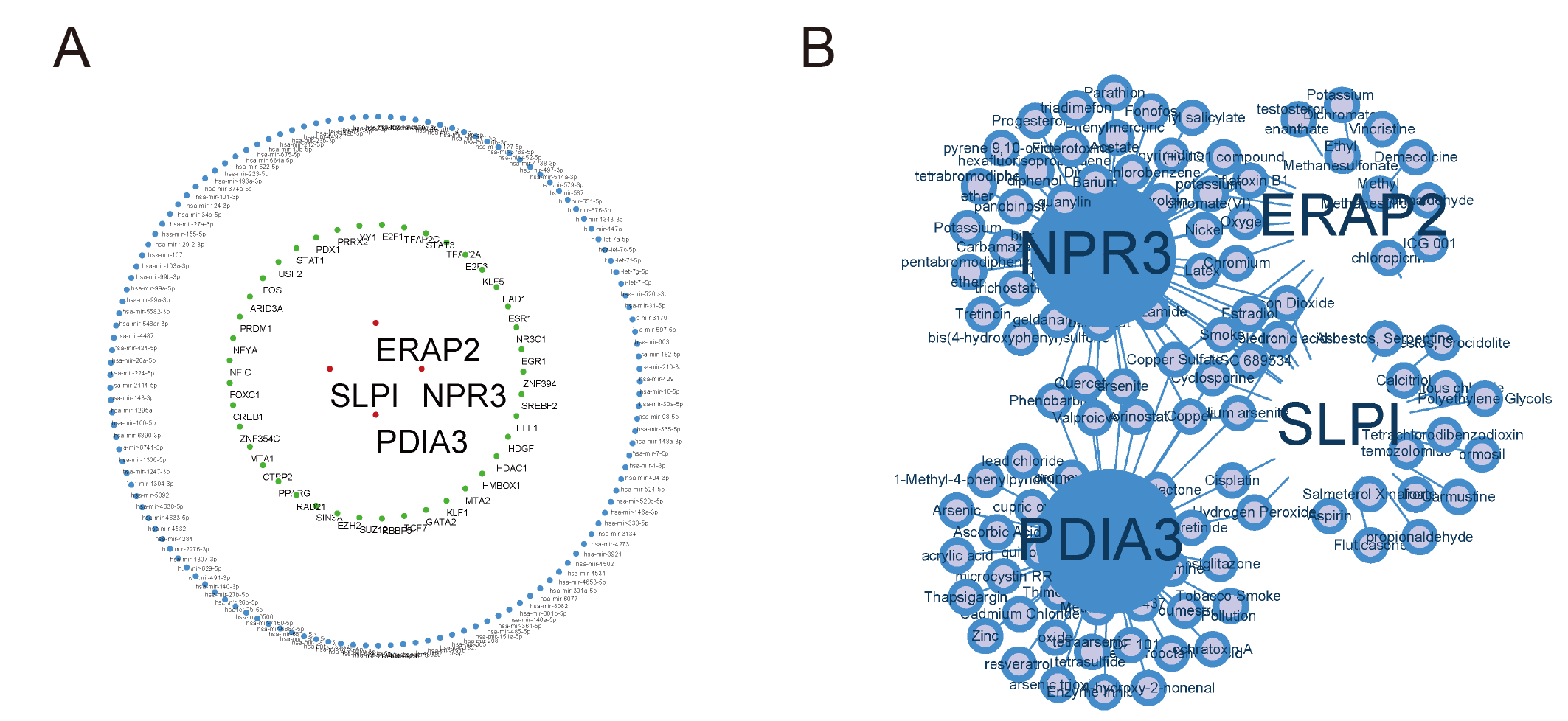

Supplement: Supplementary file 1 [file Image3.TIF]

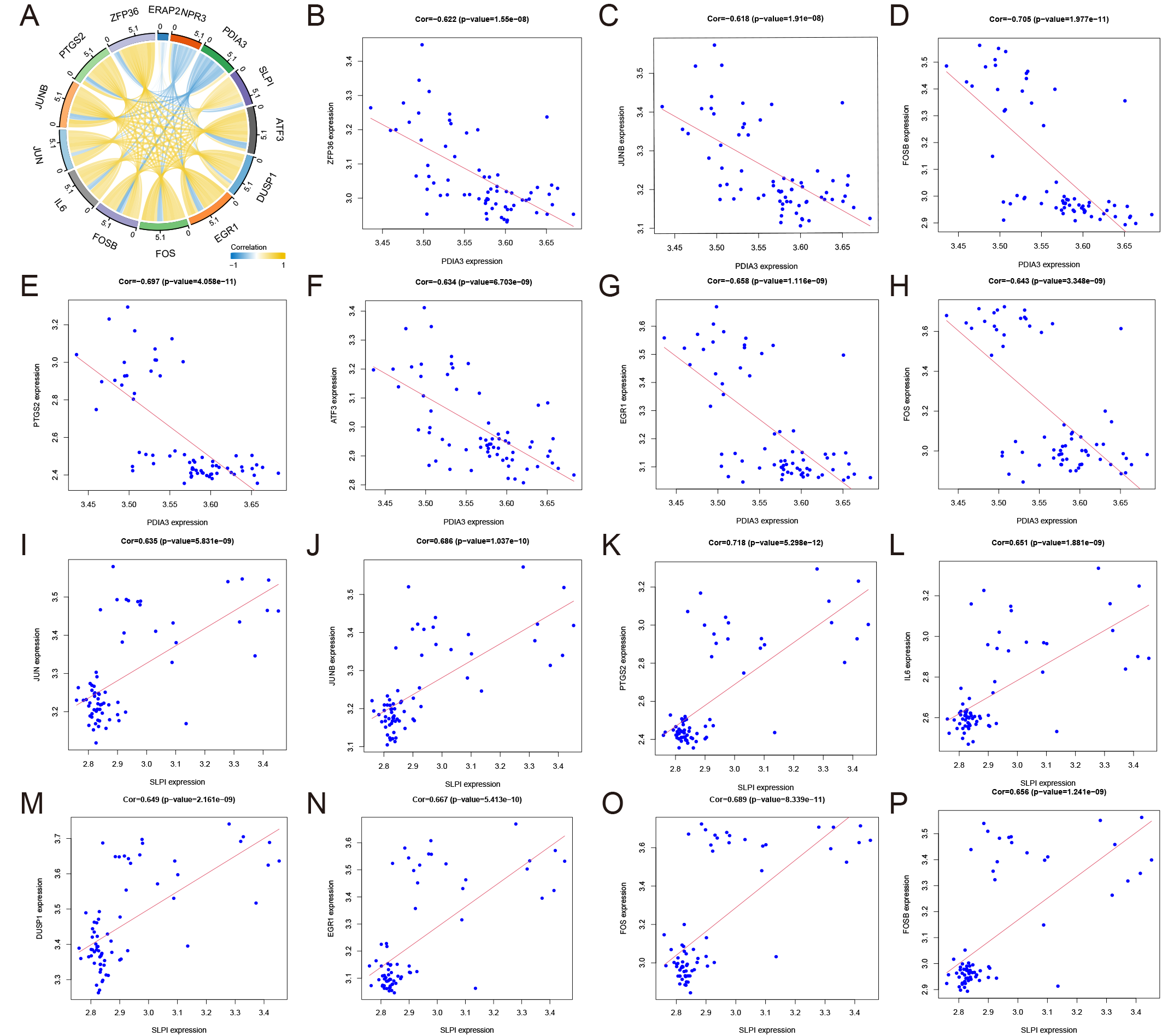

Supplement: Supplementary file 2 [file Image4.TIF]

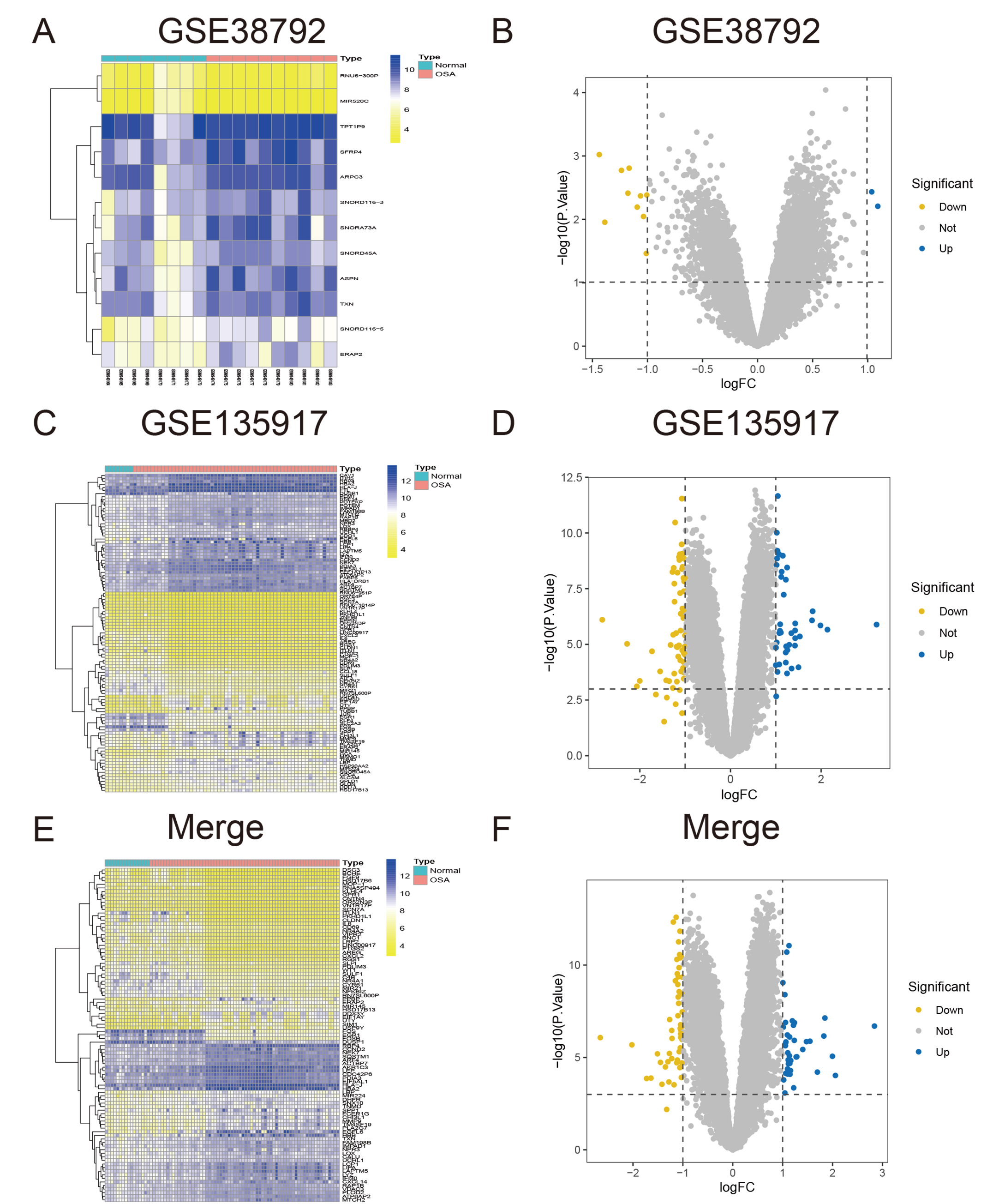

Supplement: Supplementary file 3 [file Image2.TIF]

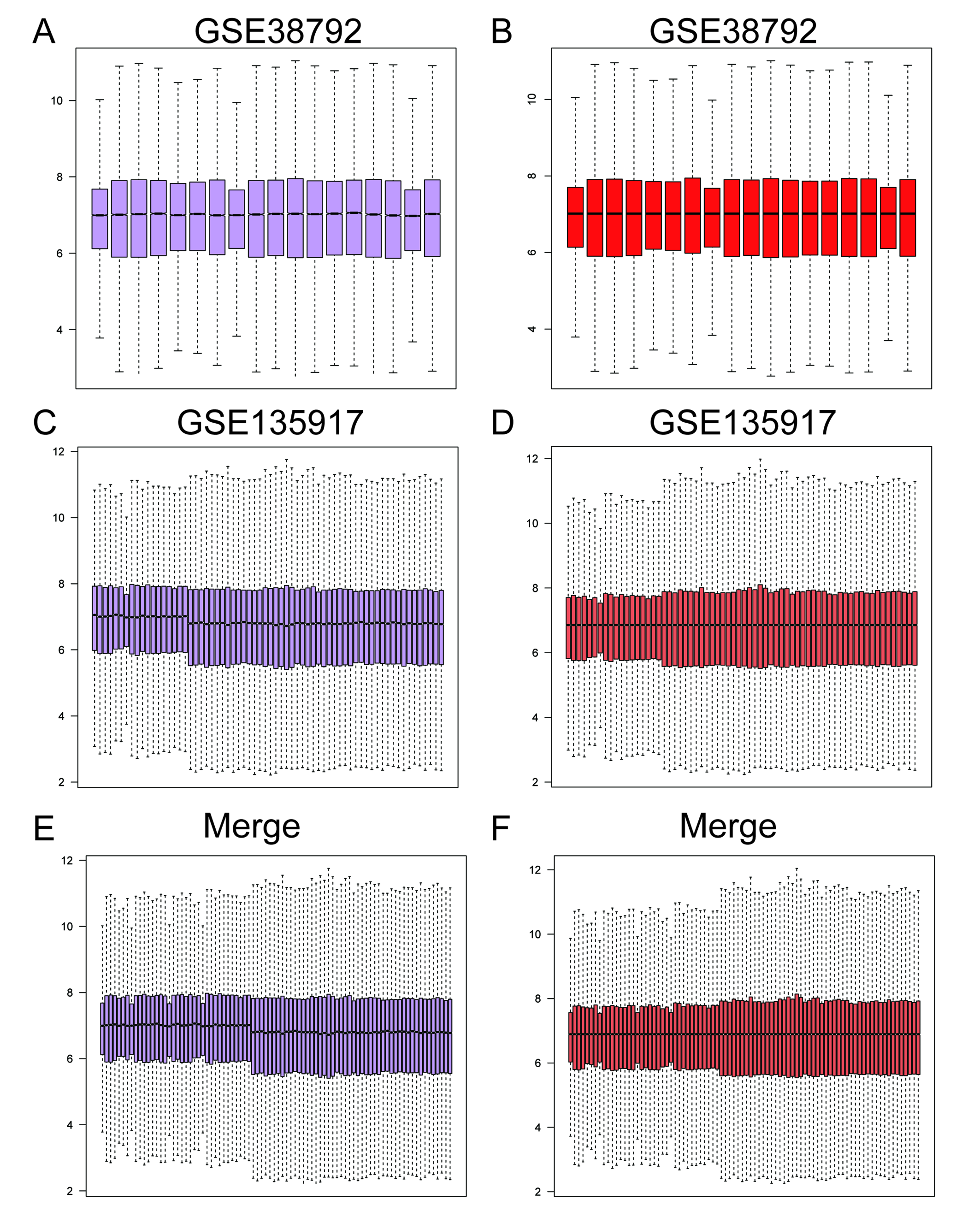

Supplement: Supplementary file 4 [file Image1.TIF]

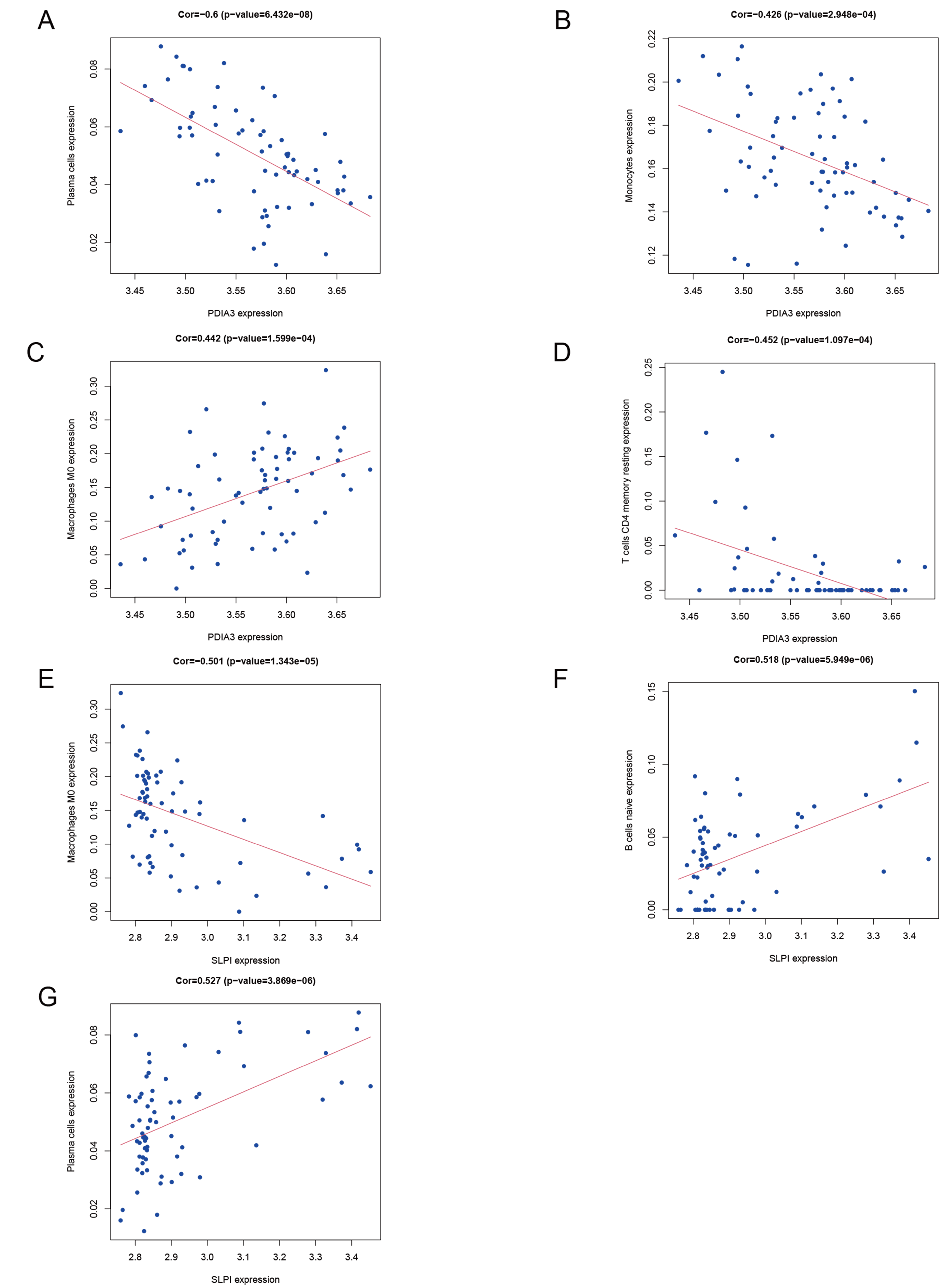

Supplement: Supplementary file 5 [file Image5.TIF]
