# Supplementary material for: Construction of a mitochondrial dysfunction related signature of diagnosed model to obstructive sleep apnea
Source: Front Genet. 2022 Nov 21;13:1056691. doi: 10.3389/fgene.2022.1056691 (PMC9714559; doi:10.3389/fgene.2022.1056691)
Supplement: Supplementary file 6 [file DataSheet1.docx]

Table.S1 Summary of data set information

| Dataset | Normal(n) | OSA(n) | | Platform | Organism | Tissue |
| --- | --- | --- | --- | --- | --- | --- |
| GSE13597 | 8 | | 58 | GPL6244 | Human | Visceral adipose |
| GSE38792 | 8 | | 10 |  |  | Subcutaneous Fat |

Table.S2 Correlation analysis between hub genes

| Gene | | cor | pvalue |
| --- | --- | --- | --- |
| SLPI | DUSP1 | 0.649 | 2.16E-09 |
|  | EGR1 | 0.667 | 5.41E-10 |
|  | FOS | 0.689 | 8.34E-11 |
|  | FOSB | 0.656 | 1.24E-09 |
|  | IL6 | 0.651 | 1.88E-09 |
|  | JUN | 0.635 | 5.83E-09 |
|  | JUNB | 0.686 | 1.04E-10 |
|  | PTGS2 | 0.718 | 5.30E-12 |
| PDIA3 | ATF3 | -0.634 | 6.70E-09 |
|  | EGR1 | -0.658 | 1.12E-09 |
|  | FOS | -0.643 | 3.35E-09 |
|  | FOSB | -0.705 | 1.98E-11 |
|  | JUNB | -0.618 | 1.91E-08 |
|  | PTGS2 | -0.697 | 4.06E-11 |
|  | ZFP36 | -0.622 | 1.55E-08 |

Table.S3 Summary of GO enrichment analysis results of 106 DEGs between clusters

| Ontology | ID | Description | GeneRatio | BgRatio | p.adjust | qvalue |
| --- | --- | --- | --- | --- | --- | --- |
| CC | GO:1904724 | tertiary granule lumen | 5/99 | 55/19717 | 9.82e04 | 8.69e04 |
| CC | GO:0060205 | cytoplasmic vesicle lumen | 9/99 | 338/19717 | 0.003 | 0.002 |
| CC | GO:0031838 | haptoglobinhemoglobin complex | 3/99 | 11/19717 | 0.002 | 0.001 |
| MF | GO:0008009 | chemokine activity | 4/93 | 49/17697 | 0.016 | 0.013 |
| MF | GO:0004252 | serinetype endopeptidase activity | 6/93 | 160/17697 | 0.017 | 0.014 |
| MF | GO:0008236 | serinetype peptidase activity | 6/93 | 182/17697 | 0.019 | 0.015 |
| CC | GO:0062023 | collagencontaining extracellular matrix | 12/99 | 406/19717 | 2.17e04 | 1.92e04 |
| BP | GO:0033002 | muscle cell proliferation | 13/97 | 239/18670 | 4.21e07 | 3.11e07 |
| BP | GO:0048660 | regulation of smooth muscle cell proliferation | 11/97 | 169/18670 | 7.17e07 | 5.30e07 |
| BP | GO:0048659 | smooth muscle cell proliferation | 11/97 | 171/18670 | 7.17e07 | 5.30e07 |
| BP | GO:0002237 | response to molecule of bacterial origin | 15/97 | 343/18670 | 4.21e07 | 3.11e07 |
| MF | GO:0001228 | DNAbinding transcription activator activity, RNA polymerase IIspecific | 12/93 | 439/17697 | 8.23e04 | 6.60e04 |

Table.S4 Summary of KEGG enrichment analysis results of 106 DEGs between clusters

| Ontology | ID | Description | GeneRatio | BgRatio | p.adjust | qvalue |
| --- | --- | --- | --- | --- | --- | --- |
| KEGG | hsa04061 | Viral protein interaction with cytokine and cytokine receptor | 5/52 | 100/8076 | 0.012 | 0.010 |
| KEGG | hsa04657 | IL17 signaling pathway | 7/52 | 94/8076 | 2.86e04 | 2.27e04 |
| KEGG | hsa04668 | TNF signaling pathway | 7/52 | 112/8076 | 4.64e04 | 3.68e04 |
| KEGG | hsa05130 | Pathogenic Escherichia coli infection | 7/52 | 197/8076 | 0.009 | 0.007 |
| KEGG | hsa04610 | Complement and coagulation cascades | 5/52 | 85/8076 | 0.009 | 0.007 |

Table.S5 Top20 | NSE | GSEA enrichment analysis list of diagnostic markers model expression

| ID | ES | NES | p | q |
| --- | --- | --- | --- | --- |
| PID_AP1_PATHWAY | 0.691 | 2.670 | 0.025 | 0.018 |
| PID_NFAT_TFPATHWAY | 0.730 | 2.579 | 0.025 | 0.018 |
| REACTOME_NGF_STIMULATED_TRANSCRIPTION | 0.743 | 2.515 | 0.025 | 0.018 |
| WP_CYTOPLASMIC_RIBOSOMAL_PROTEINS | 0.604 | 2.422 | 0.025 | 0.018 |
| REACTOME_EUKARYOTIC_TRANSLATION_ELONGATION | 0.598 | 2.413 | 0.025 | 0.018 |
| REACTOME_NUCLEAR_EVENTS_KINASE_AND_TRANSCRIPTION_FACTOR_ACTIVATION_ | 0.642 | 2.408 | 0.025 | 0.018 |
| KEGG_RIBOSOME | 0.599 | 2.401 | 0.025 | 0.018 |
| KEGG_COMPLEMENT_AND_COAGULATION_CASCADES | 0.611 | 2.353 | 0.025 | 0.018 |
| WP_COMPLEMENT_ACTIVATION | 0.790 | 2.342 | 0.025 | 0.018 |
| PID_TCR_CALCIUM_PATHWAY | 0.730 | 2.325 | 0.025 | 0.018 |
| BIOCARTA_LECTIN_PATHWAY | 0.904 | 2.305 | 0.025 | 0.018 |
| REACTOME_COMPLEMENT_CASCADE | 0.603 | 2.296 | 0.025 | 0.018 |
| WP_COMPLEMENT_AND_COAGULATION_CASCADES | 0.612 | 2.278 | 0.025 | 0.018 |
| BIOCARTA_COMP_PATHWAY | 0.781 | 2.252 | 0.025 | 0.018 |
| BIOCARTA_CLASSIC_PATHWAY | 0.852 | 2.248 | 0.025 | 0.018 |
| REACTOME_INITIAL_TRIGGERING_OF_COMPLEMENT | 0.687 | 2.233 | 0.025 | 0.018 |
| REACTOME_RESPONSE_OF_EIF2AK4_GCN2_TO_AMINO_ACID_DEFICIENCY | 0.542 | 2.221 | 0.025 | 0.018 |
| WP_PHOTODYNAMIC_THERAPYINDUCED_AP1_SURVIVAL_SIGNALING | 0.615 | 2.217 | 0.025 | 0.018 |
| BIOCARTA_CCR5_PATHWAY | 0.781 | 2.163 | 0.025 | 0.018 |
| PID_REG_GR_PATHWAY | 0.543 | 2.156 | 0.025 | 0.018 |
| KEGG_PROTEASOME | 0.701 | -2.422 | 0.025 | 0.018 |
| KEGG_LYSOSOME | 0.573 | -2.404 | 0.025 | 0.018 |
| REACTOME_APC_C_MEDIATED_DEGRADATION_OF_CELL_CYCLE_PROTEINS | 0.588 | -2.362 | 0.025 | 0.018 |
| REACTOME_APC_C_CDH1_MEDIATED_DEGRADATION_OF_CDC20_AND_OTHER_APC_C_CDH1_TARGETED_PROTEINS_IN_LATE_MITOSIS_EARLY_G1 | 0.602 | -2.317 | 0.025 | 0.018 |
| REACTOME_UCH_PROTEINASES | 0.581 | -2.316 | 0.025 | 0.018 |
| REACTOME_SWITCHING_OF_ORIGINS_TO_A_POST_REPLICATIVE_STATE | 0.572 | -2.306 | 0.025 | 0.018 |
| REACTOME_METABOLISM_OF_POLYAMINES | 0.626 | -2.304 | 0.025 | 0.018 |
| REACTOME_ORC1_REMOVAL_FROM_CHROMATIN | 0.595 | -2.277 | 0.025 | 0.018 |
| REACTOME_DECTIN_1_MEDIATED_NONCANONICAL_NF_KB_SIGNALING | 0.608 | -2.264 | 0.025 | 0.018 |
| REACTOME_NEGATIVE_REGULATION_OF_NOTCH4_SIGNALING | 0.631 | -2.260 | 0.025 | 0.018 |
| KEGG_GLYCOSYLPHOSPHATIDYLINOSITOL_GPI_ANCHOR_BIOSYNTHESIS | 0.733 | -2.257 | 0.025 | 0.018 |
| REACTOME_DEGRADATION_OF_GLI1_BY_THE_PROTEASOME | 0.612 | -2.250 | 0.025 | 0.018 |
| REACTOME_RETROGRADE_TRANSPORT_AT_THE_TRANS_GOLGI_NETWORK | 0.638 | -2.246 | 0.025 | 0.018 |
| REACTOME_STABILIZATION_OF_P53 | 0.614 | -2.244 | 0.025 | 0.018 |
| REACTOME_INTRA_GOLGI_AND_RETROGRADE_GOLGI_TO_ER_TRAFFIC | 0.501 | -2.238 | 0.025 | 0.018 |
| REACTOME_DEFECTIVE_CFTR_CAUSES_CYSTIC_FIBROSIS | 0.602 | -2.231 | 0.025 | 0.018 |
| REACTOME_SEPARATION_OF_SISTER_CHROMATIDS | 0.504 | -2.229 | 0.025 | 0.018 |
| REACTOME_DNA_REPLICATION | 0.527 | -2.226 | 0.025 | 0.018 |
| REACTOME_CROSS_PRESENTATION_OF_SOLUBLE_EXOGENOUS_ANTIGENS_ENDOSOMES_ | 0.630 | -2.225 | 0.025 | 0.018 |
| REACTOME_DNA_REPLICATION_PRE_INITIATION | 0.558 | -2.223 | 0.025 | 0.018 |
